# Artificial intelligence algorithm for detecting myocardial infarction using six-lead electrocardiography

**DOI:** 10.1038/s41598-020-77599-6

**Published:** 2020-11-24

**Authors:** Younghoon Cho, Joon-myoung Kwon, Kyung-Hee Kim, Jose R. Medina-Inojosa, Ki-Hyun Jeon, Soohyun Cho, Soo Youn Lee, Jinsik Park, Byung-Hee Oh

**Affiliations:** 1 Medical Research and Development Center, Bodyfriend, Seoul, South Korea; 2 Medical Technology Laboratory, Bodyfriend, Seoul, South Korea; 3 Department of Emergency Medicine, Mediplex Sejong Hospital, 20, Gyeyangmunhwa-ro, Gyeyang-gu, Incheon, South Korea; 4 Artificial Intelligence and Big Data Research Center, Sejong Medical Research Institute, Bucheon, South Korea; 5 Medical Research Team, Medical AI, Seoul, South Korea; 6 Division of Cardiology, Department of Internal Medicine, Cardiovascular Center, Mediplex Sejong Hospital, 20, Gyeyangmunhwa-ro, Gyeyang-gu, Incheon, South Korea; 7 Division of Preventive Cardiology, Department of Cardiovascular Medicine, Mayo Clinic, Rochester, MN USA

**Keywords:** Cardiology, Information technology

## Abstract

Rapid diagnosis of myocardial infarction (MI) using electrocardiography (ECG) is the cornerstone of effective treatment and prevention of mortality; however, conventional interpretation methods has low reliability for detecting MI and is difficulty to apply to limb 6-lead ECG based life type or wearable devices. We developed and validated a deep learning-based artificial intelligence algorithm (DLA) for detecting MI using 6-lead ECG. A total of 412,461 ECGs were used to develop a variational autoencoder (VAE) that reconstructed precordial 6-lead ECG using limb 6-lead ECG. Data from 9536, 1301, and 1768 ECGs of adult patients who underwent coronary angiography within 24 h from each ECG were used for development, internal and external validation, respectively. During internal and external validation, the area under the receiver operating characteristic curves of the DLA with VAE using a 6-lead ECG were 0.880 and 0.854, respectively, and the performances were preserved by the territory of the coronary lesion. Our DLA successfully detected MI using a 12-lead ECG or a 6-lead ECG. The results indicate that MI could be detected not only with a conventional 12 lead ECG but also with a life type 6-lead ECG device that employs our DLA.

## Introduction

Myocardial infarction (MI) is a major global health care burden, with an estimated 7.29 million MIs and 110.55 million prevalent cases of ischemic heart disease (IHD) annually^1,2^. There are an estimated 8.92 million deaths due to IHD annually, making IHD the leading cause of death worldwide^2^. Approximately 15 million patients per year in the United States and Europe are admitted to the emergency department with chest pain or other symptoms suggestive of MI^3^. Rapid identification of MI is critical for the initiation of effective evidence-based medical management and prevention of mortality^4,5^. Any delays in treating patients with MI affect mortality and increase the risk of death, which is highest within the first hours from chest pain onset^5,6^.

In addition to the clinical symptoms, electrocardiography (ECG) and measurement of cardiac troponins are the current diagnostic cornerstone^4^. Although cardiac troponins are important biomarkers for the diagnosis of MI, the laboratory test is invasive and requires specialized equipment, cost, and infrastructures, such as trained medical staff for sampling blood and performing assays, and a hematology analyzer with biochemical reagents. For this reason, cardiac troponins are difficult to use in remote healthcare monitoring or developing countries. ECG is a non-invasive test that allows continuous and remote monitoring. However, ECG by itself is often insufficient to diagnose an MI, as ST-segment deviation may be observed in other conditions such as acute pericarditis, left ventricular hypertrophy, left bundle-branch block, Brugada syndrome, and early repolarizations^7^. Because of this, the rule-based automatic ECG reading in a conventional ECG machine has low reliability for detecting MI, and cardiologists are, therefore, unable to diagnose MI with an ECG alone^8,9^. Furthermore, conventional methods that can be used to diagnose MI by utilizing information from a 12-lead ECG are difficult to apply to a limb 6-lead ECG based life type or wearable device^10^.

Deep learning is a type of artificial intelligence approach that extracts and uses meaningful patterns from complex digital data and has recently been used to analyze ECGs for diagnosing an arrhythmia, heart failure, left ventricular hypertrophy, valvular heart disease, age, and sex^11–16^. To develop a reliable MI detecting method based on ECG, we used deep learning. We hypothesized that a deep-learning-based algorithm (DLA) could effectively detect MI using ECG. To test this hypothesis, we developed and validated a DLA for detecting MI using a 12- and a 6-lead ECG.

## Results

The study population included 425,066 ECGs of 292,152 patients. Of them, 412,461 ECGs of 283,878 patients in hospital A who did not undergo CAG were used to develop VAE. From 6543, 37 were excluded, 5205 were used as development data, and 1301 for internal validation. A total of 9536 ECGs from 5205 patients were used as development data, and 1301 ECGs from 1301 patients were used as internal validation data. Data from 1768 ECGs of 1768 patients from hospital B were used as external validation data. Of the patients used for development, internal validation, and external validation, the number of MI patients was 2081, 200, and 225, respectively. The baseline characteristics of the study population are shown in Table 1.

**Table 1:** Baseline characteristics.

	Hospital A: development and internal validation data			Hospital B: external validation data			*P*
	Non-MI (n = 5466)	MI (n = 1040)	*P*	Non-MI (n = 1543)	MI (n = 225)	*P*	
Age, year, mean (SD)	64.26 (11.37)	63.84 (12.29)	0.290	63.11 (11.82)	62.29 (13.58)	0.342	< 0.001
Male, n (%)	2191 (40.1)	278 (26.7)	< 0.001	588 (38.1)	67 (29.8)	0.019	0.505
Heart rate, bpm, mean (SD)	73.76 (18.58)	77.83 (19.62)	< 0.001	73.06 (18.37)	78.21 (19.43)	< 0.001	0.169
**Electrocardiographic feature**							
PR interval, ms, mean (SD)	172.28 (28.96)	174.97 (34.54)	0.011	172.17 (27.67)	171.71 (33.28)	0.830	0.464
QT interval, ms, mean (SD)	− 0.12 (0.92)	− 0.15 (1.02)	0.279	− 0.07 (0.97)	− 0.17 (1.08)	0.176	0.143
QRS duration, ms, mean (SD)	− 0.06 (0.91)	0.01 (0.93)	0.014	− 0.03 (0.97)	0.00 (0.79)	0.673	0.263
QTc, mean (SD)	− 0.20 (0.91)	0.03 (0.99)	< 0.001	− 0.17 (0.95)	0.06 (0.98)	0.001	0.241
P axis, mean (SD)	45.02 (27.69)	47.16 (28.03)	0.032	42.14 (29.84)	44.00 (32.31)	0.407	< 0.001
R axis, mean (SD)	0.05 (0.88)	0.01 (1.01)	0.194	0.07 (0.92)	− 0.10 (1.07)	0.011	0.792
T axis, mean (SD)	− 0.12 (0.85)	0.08 (1.02)	< 0.001	− 0.11 (0.87)	0.12 (1.12)	< 0.001	0.777

**Figure 1:**
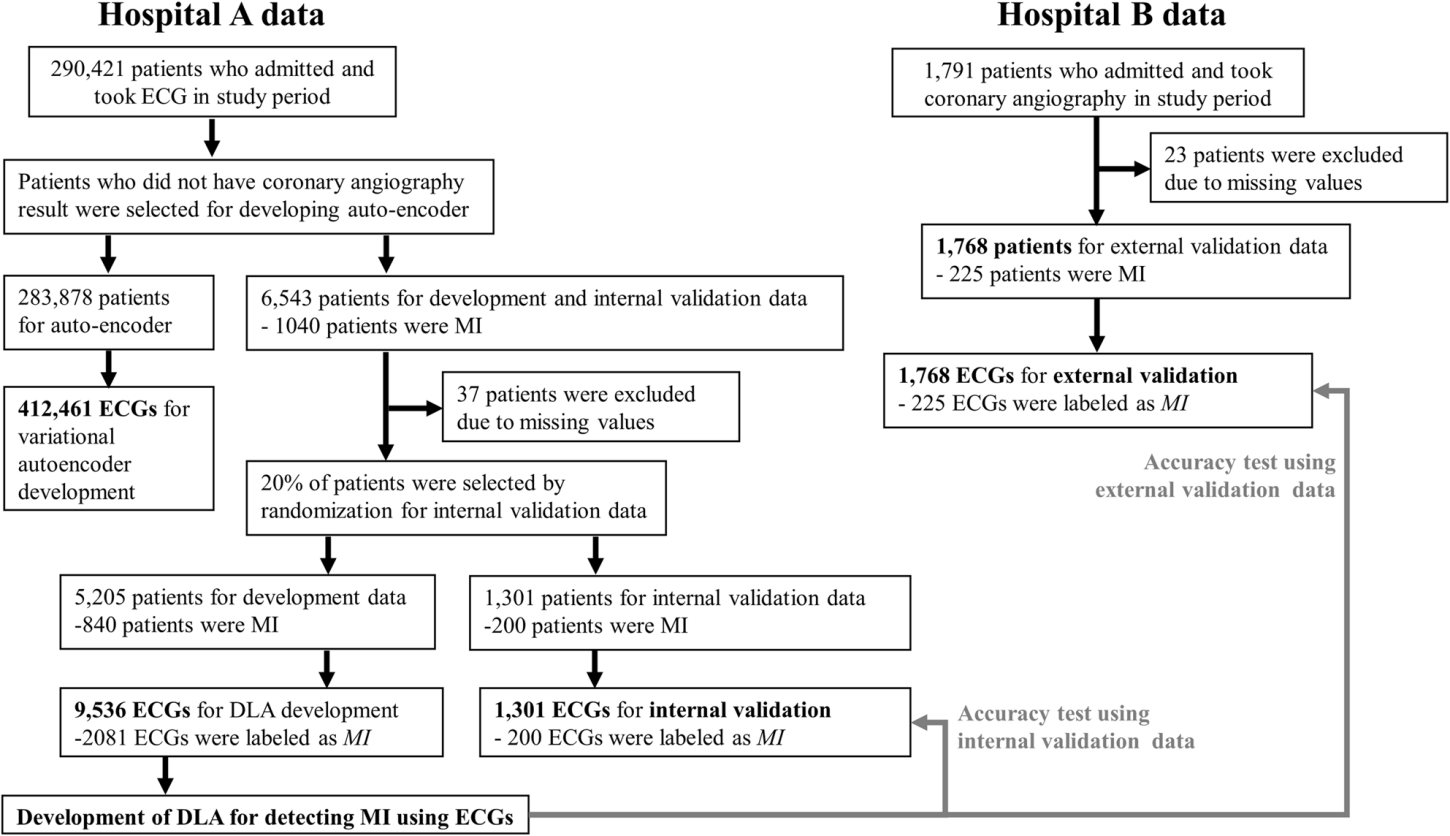
Study flowchart. *DLA* deep learning-based algorithm, *ECG* electrocardiography, *MI* myocardial infarction.

As shown in Fig. 1 and Table 1, the prevalence of MI patients was 16.1% (840/5205), 15.3% (200/1301), and 12.7% (225/1768) in development, internal, and external validation, respectively. The percentage of MI ECG in the entire ECG series was 21.8% (2081/9536), 15.3%, and 12.7% in development, internal, and external validation, respectively. First, as more ECGs were taken for MI patients than non-MI patients for evaluating progression and we used all the ECGs within the coronary angiography for the development dataset, the proportion of MI ECG was higher than that of MI patients in the development dataset. Secondly, the development and internal validation data were from hospital A (cardiovascular specialty teaching hospital) and the external validation data were from hospital B (community general hospital). Because of this, the incidence of MI from hospital A (development and internal validation data) was higher than that of hospital B (external validation data).

**Figure 2:**
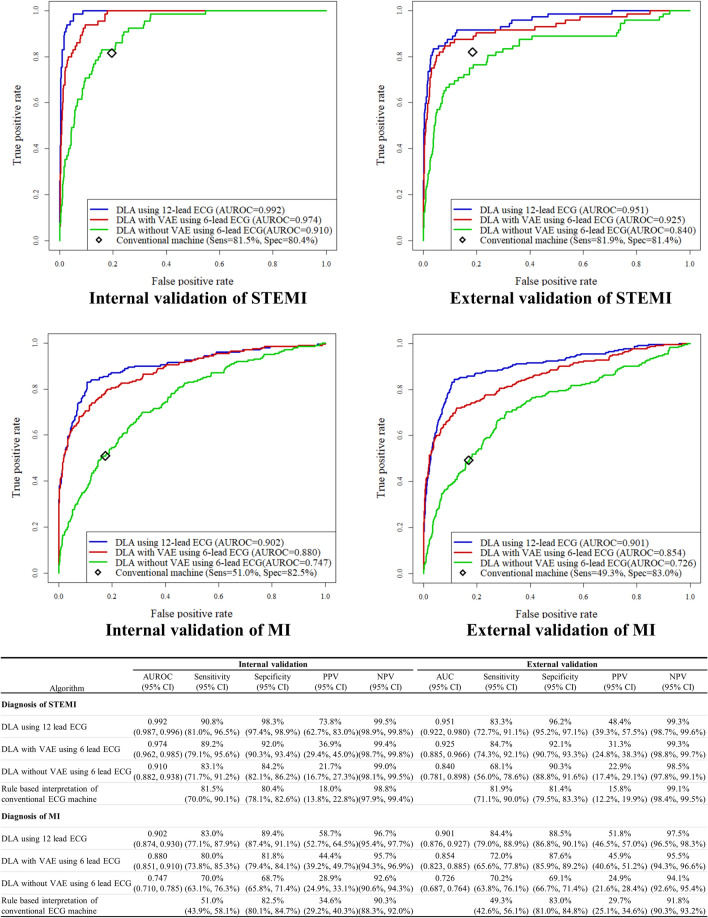
Performances of artificial intelligence algorithms for detecting myocardial infarction. *AUC* area under the receiver operating characteristics curve, *DLA* deep learning-based algorithm, *ECG* electrocardiography, *MI* myocardial infarction, *NPV* negative predictive value, *Sens* sensitivity, *Spec* specificity, *STEMI* ST-segment elevation myocardial infarction, *PPV* positive predictive value, *VAE* variational autoencoder. These performances were calculated by using the operating point at Youden J statistics of development data.

For the endpoint (confirmed MI using information from CAG and lab test within 24 h after ECG), the AUROC of the DLA using a 12-lead ECG was 0.902 (95% confidence interval: 0.874–0.930) and 0.901 (0.876–0.927) during internal and external validation, respectively. The AUROC of the 6-lead DLA with VAE was 0.880 (0.851–0.910) and 0.854 (0.823–0.885) during internal and external validation, respectively. The AUROC of the 6-lead DLA without VAE was 0.747 (0.710–0.785) and 0.726 (0.687–0.764) during internal and external validation, respectively. The sensitivity, specificity, PPV, and NPV of the DLA using 12-lead ECG was 83.0%, 89.4%, 58.7%, and 96.7%, respectively, in internal validation data. In the external validation data, the DLAs’ sensitivity, specificity, NPV, and PPV were 84.4%, 88.5%, 51.8%, and 97.5%, respectively. As shown in Fig. 2, the AUROCs of DLA using a 12-lead ECG for detecting ST-segment elevation MI (STEMI) were 0.992 and 0.951 in internal and external validation. DLA with VAE using a 6-lead ECG also achieved AUROCs of 0.974 and 0.925 for detecting STEMI in internal and external validation, respectively. As shown in Fig. 2, DLA using a 12-lead ECG and DLA with VAE using a 6-lead ECG outperformed rule-based interpretation of conventional ECG machine.

**Figure 3:**
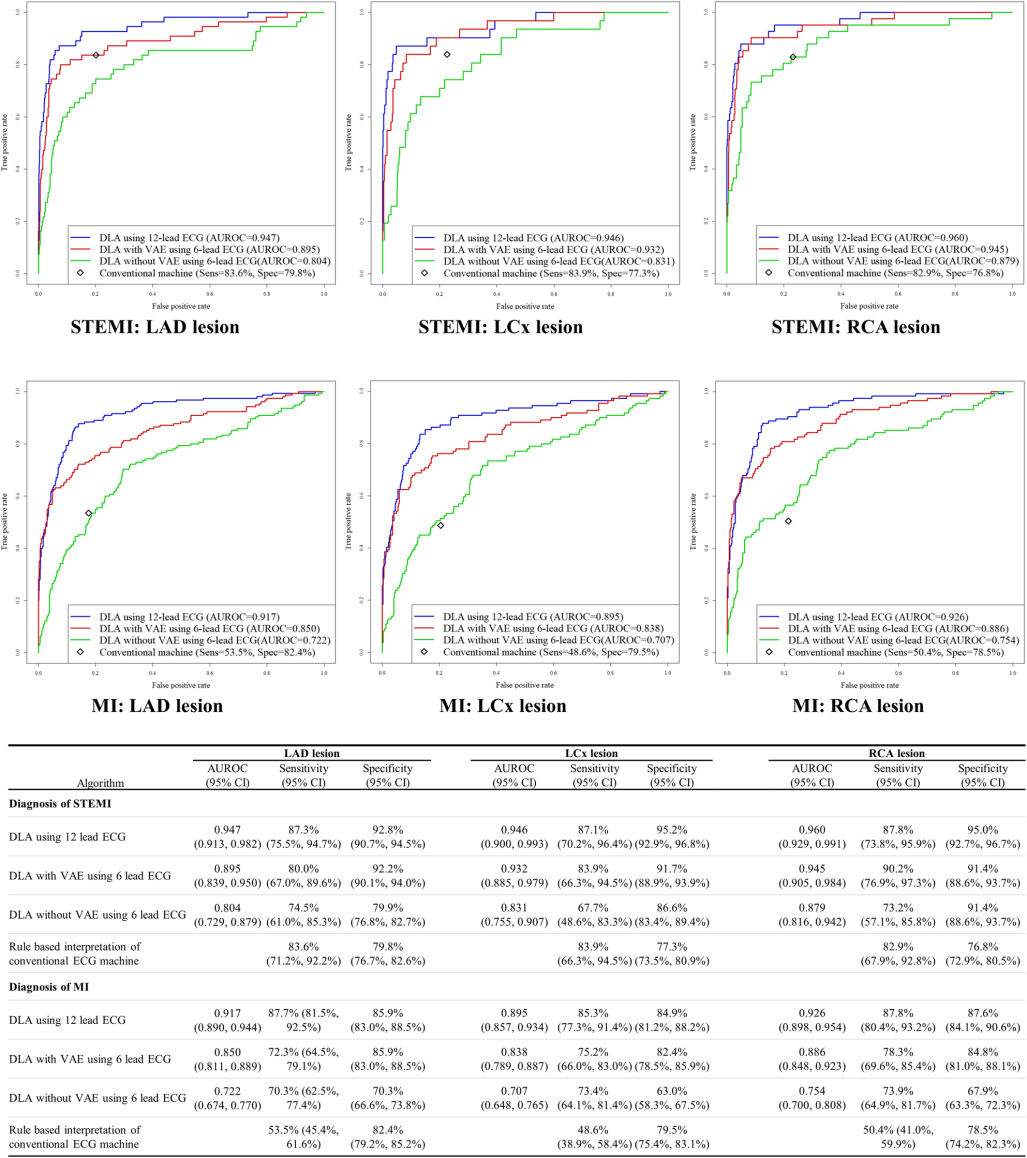
Performances of artificial intelligence algorithms for detecting MI by coronary artery lesion. *AE* auto-encoder, *AUC* area under the receiver operating characteristics curve, *DLA* deep learning-based algorithm, *ECG* electrocardiography, *LAD* left anterior descending coronary artery, *LCx* left circumflex coronary artery, *MI* myocardial infarction, *NPV* negative predictive value, *Sens* sensitivity, *Spec* specificity, *STEMI* ST-segment elevation myocardial infarction, *PPV* positive predictive value, *RCA* right coronary artery. These performances were calculated by using the operating point at Youden J statistics of development data.

**Figure 4:**
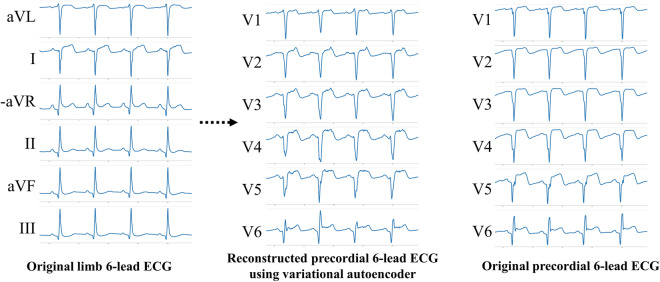
Reconstructed precordial 6-lead ECG using limb 6-lead ECG by auto-encoder and original precordial 6-lead ECG. *DLA* deep learning-based algorithm, *ECG* electrocardiography, *MI* myocardial infarction.

For the internal and external validation dataset, we confirmed the performance of DLAs stratified by coronary artery territory. As shown in Fig. 3, the performances of the 12-lead ECG DLAs were similar when assessed by the coronary lesion. When using limb 6-lead ECG, DLA with AE showed preserved performance but DLA without AE showed poor performance. As shown in Fig. 4, AE reconstructed precordial 6-lead ECG using limb 6-lead ECG. The mean squared errors in internal and external validation data were 0.015 and 0.014, respectively.

**Figure 5:**
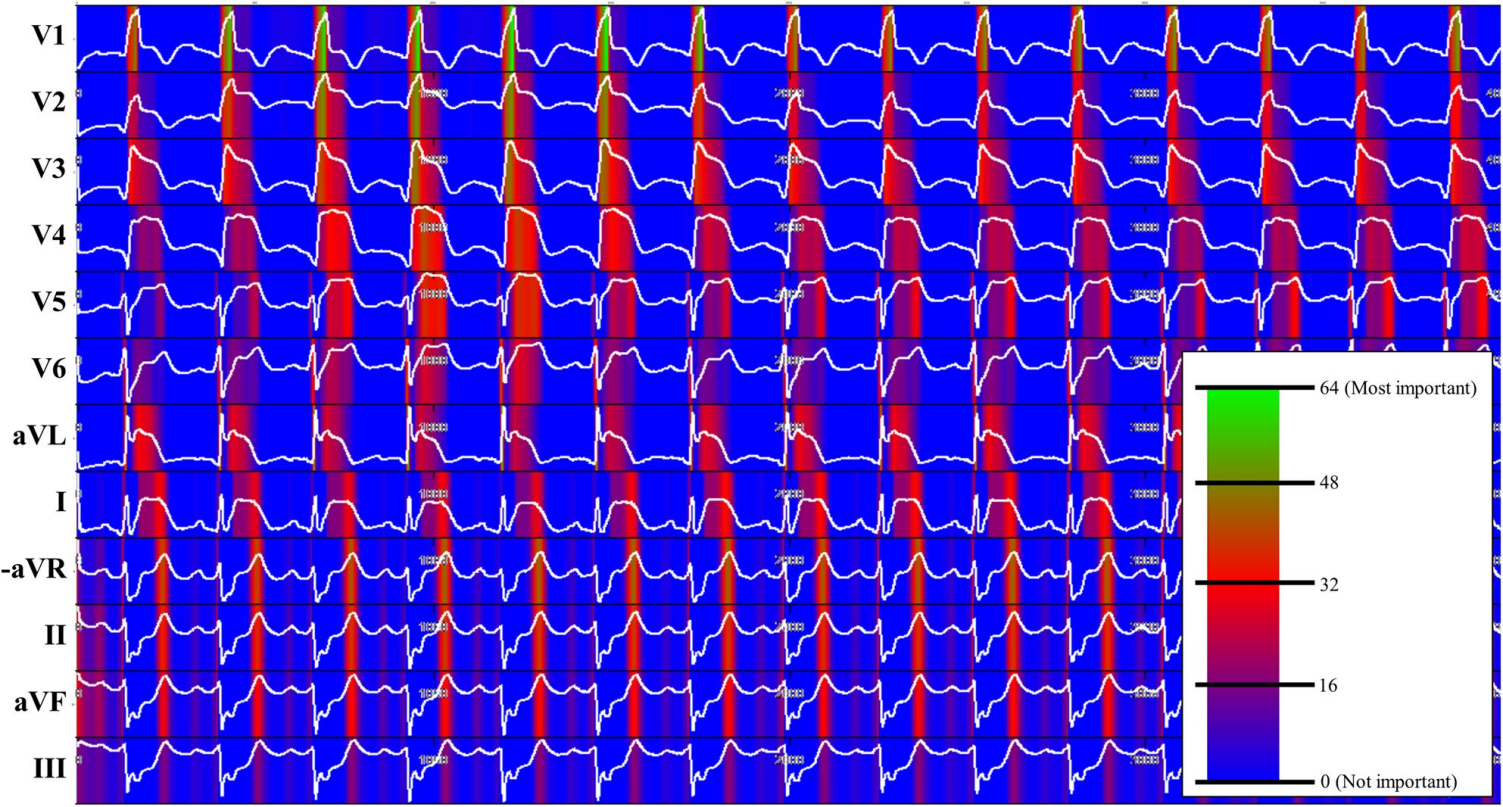
Sensitivity map of DLA for detecting MI. *DLA* deep learning-based algorithm, *MI* myocardial infarction.

As shown in Fig. 5, the sensitivity map shows that the DLA focused mostly on the ST segment to detect MI. The DLA also focused on T-wave, but the significances of these regions were lower than that of the QRS complex. We have provided several sensitivity maps of MI ECGs as Supplemental Material.

## Methods

This study was approved by the Institutional Review Boards of Sejong General Hospital (2019-0239) and Mediplex Sejong Hospital (2019-083) and all methods were carried out in accordance with relevant guidelines and regulations. Clinical data, including digitally stored ECGs, coronary intervention reports, laboratory results, emergency department, and admission notes, and epidemiologic information were used. The need for informed consent was waived because of the retrospective nature of the study using fully anonymized ECG and health data, with minimal potential for harm.

### ECG data and endpoint

Predictor variables are ECG, age, and sex. Digitally stored 12-lead ECG data, amounting to 5000 numbers for each lead was recorded over 10 s (500 Hz). We removed 1 s each at the beginning and end of ECG because these areas have more artifacts than other parts. Because of this, the length of each was 8 s (4000 numbers). We made a dataset using the entire 12-lead ECG data. We also used partial datasets from 12-lead ECG data, limb 6-lead (aVL, I, − aVR, II, aVF, and III). We selected these sets of leads because they can easily be recorded with wearable devices, pads, and other daily living devices while in contact with the extremities^10^. Consequently, when we developed and validated an algorithm using 12-lead ECGs, we used a dataset of 2-dimensional (2D) data of 12 × 4000 numbers. To make the input 2D ECG data, we rearranged the data in the order of V1, V2, V3, V4, V6, aVL, I, -aVR, II, aVF, and III. The convolutional neural network (CNN), a method of deep learning, is a well-known architecture for learning 2D image data^16^. In the same manner, when we developed and validated an algorithm using a 6-lead; we used datasets that were 6 × 4000.

The primary endpoints were type 1 and 2 MIs defined in the fourth universal definition of MI^17^. Four cardiologists manually reviewed the patients’ medical records and results of cardiac enzymes, coronary angiography (CAG), and echocardiography to label the occurrence of an MI at the time of CAG. A cardiologist labeled the development dataset, and three cardiologists not involved in the development data only labeled the internal and external validation data. After labeling the validation data, we used a voting system to render a decision when an agreement from the cardiologists is not reached. Cardiologists labeled MI using the gold standard of CAG findings such as a primary coronary event such as plaque erosion and/or rupture, fissuring, or dissection, and supply/demand imbalance such as coronary spasm and elevation of cardiac troponins, which were above the 99th percentile upper reference limit at the time of CAG. With regard to development, internal validation, and external validation, the ECGs that were acquired within 24 h before MI CAG were labeled as MI. The ECGs that were acquired within 24 h before non-MI CAG were labeled as non-MI. We only used ECGs acquired less than 24 h before the initiation of CAG.

### Development of a deep-learning-based algorithm

**Figure 6:**
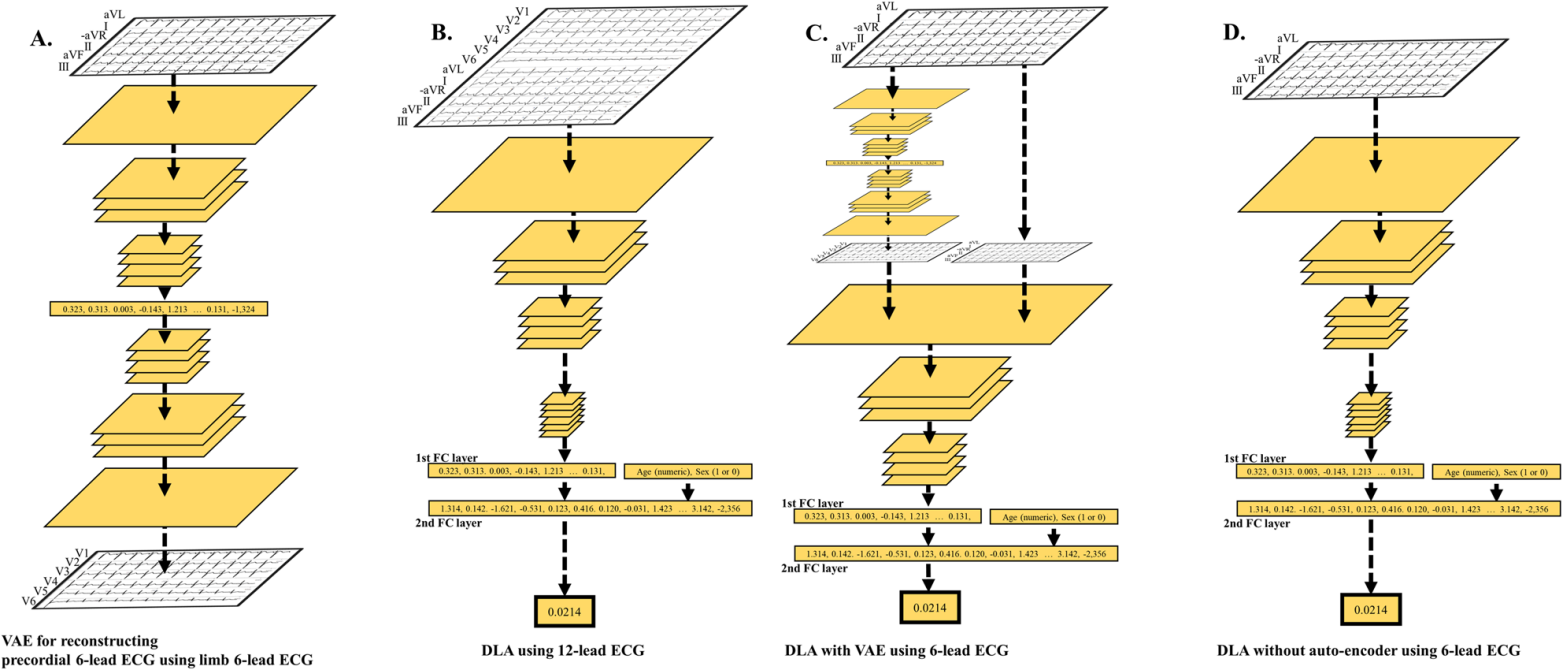
Architecture of variational autoencoder and deep learning-based algorithm for detecting MI using a 12-lead or a 6-lead ECG. *DLA* deep learning-based algorithm, *ECG* electrocardiography, *FC* fully connected, *MI* myocardial infarction, *VAE* variational autoencoder.

The DLA was made using many hidden layers of neurons^16^. As a block with seven stages, there are two convolution layers, two batch normalizations, one max pooling, and one dropout layer repeated (Fig. 6)^18,19^. The last convolutional layer of the CNN connected to a flattened layer, fully connected to the 1-dimensional layer and composed of 128 nodes. The input layer of epidemiology data (age and sex) was concatenated with the 1-dimensional layer. There were two fully connected 1-dimensional layers after the flattened layer, and the second fully connected 1-dimensional layer was connected to the output layer, which was composed of 1 node. The output values of the output node represented the possibility of an MI, and the output node used a sigmoid function as an activation function because the output of the sigmoid function was between 0 and 1. We used TensorFlow (Google LLC, Mountain View, CA USA) as the backend and conducted the experiment with Python (version 3.5.2; Python Software Foundation, Beaverton, OR USA)^20^. We conducted additional experiments for DLA using limb 6-lead ECG. For developing and validating the DLA for a 6-lead ECG, we changed the size of the filter and convolutional layers for adjusting the shapes of input datasets. The number of filters, the maximal pooling, and the fully connected layers were the same as in the architecture of DLA using 12-lead ECG.

For enhancing the performance of DLA using limb 6-lead ECG, we developed a variational autoencoder (VAE), which is a type of artificial neural network that is used for image reconstruction and extracted features^21^. Our VAE reconstructed a precordial 6-lead ECG using a limb 6-lead ECG. The VAE contained an encoder and decoder. The encoder and decoder comprised 6 CNN layers each, and the encoder and decoder were connected by a 1-dimensional dense layer. After developing the VAE, we adopted it to develop an enhanced DLA using 6-lead ECG. That is, when we input limb 6-lead ECG into DLA, the DLA reconstructed the precordial 6-lead ECG, and then it used all information (limb 6-lead ECG and reconstructed precordial 6-lead ECG) for detecting MI. The concept is illustrated in Fig. 6.

### Development, validation, and AE datasets

Data from hospital A was used for VAE, development, and internal validation. Using data from hospital A, we identified patients with at least one standard digital, 10-s, 12-lead ECG acquired in the supine position within the study period (October 2016–September 2019). The patients with at least one coronary artery evaluation acquired from conventional coronary angiography (CAG) obtained within 24 h after the index ECG were selected for the development and validation dataset. The patients without CAG results were selected for VAE development, as shown in Fig. 1. We excluded subjects with missing demographics, electrocardiographic, cardiac enzyme results, or CAG information from the development and internal validation dataset. As shown in Fig. 1, the patients were randomly split into algorithm development (80%) and internal validation (20%) datasets, exclusively. Data from hospital B were used for external validation, which confirmed that the developed DLA was robust across diverse hospitals. Hospital A was a cardiovascular teaching hospital and hospital B was a community general hospital. We identified the patients who were admitted to Hospital B in the study period (March 2017–September 2019) and who had at least one ECG and at least one CAG result within 24 h of the index ECG. We also excluded subjects in hospital B with missing values. In the development data, we took several ECGs within 24 h prior to coronary angiography and used all the ECGs as the development dataset. Because the purpose of the validation data was to assess the accuracy of the algorithm, we only used one ECG from each patient for the internal and external validation datasets—the most recent ECG to their CAG in the study period. The datasets for development, validation, and VAE were exclusively divided.

### Statistical analysis

At each input (ECG, age, and sex) of the validation data, the DLA calculated the possibility of MI in the range from 0 (*non-MI*) to 1 (*MI*). To confirm the performance of the DLA, we compared the possibility calculated by the DLA with the endpoint (*MI* of *non-MI*) of each ECG. For this, we used the area under the receiver operating characteristics curve (AUROC) to measure the performance of the model. We used rule-based dictation of conventional ECG machines (PageWriter, Philips) as comparative methods. And we confirmed the performance of DLA based on the location of the coronary lesion from medical records. We used the dictation of the ECG machine, which was saved in each ECG raw data file. As the output values of the output node were between 0 and 1, we needed a cutoff value for calculating the sensitivity, specificity, positive predictive value (PPV), and negative predictive value (NPV). We selected the cutoff point (cutoff value) using Youden J statistics in the development data. We applied the cutoff point to internal and external validation data to calculate sensitivity, specificity, negative predictive value, and positive predictive value. Sensitivity, specificity, PPV, and NPV were confirmed at the operating point from Youden J statistics in the development data^22^. Exact 95% confidence intervals (CIs) were used for all measures of diagnostic performance except for AUROC. The CI for AUROC was determined based on Sun and Su optimization of the De-long method, using the pROC package in R (The R Foundation, Vienna, Austria; www.r-project.org). Statistical significance for the differences in patient characteristics was defined as a 2-sided *P* value of less than 0.001. Measures of the diagnostic performance were summarized using 2-sided 95% CIs. Analyses were computed using R software, version 3.4.2.

### Visualizing using sensitivity map

To understand the developed DLA and make a comparison with existing medical knowledge of MI, it was important to identify which regions had significant effects on the decision of the DLA. We employed a sensitivity map using the saliency method and used it to visualize the ECG regions used by the DLA to detect MI^23^. The map was computed using the first-order gradients of the classifier probabilities concerning the input signals. If the probability of a classifier was sensitive to a specific region of the signal, the region would be considered as significant in the model. We used a gradient-weighted class activation map (Grad-CAM) for visualization^23^. Grad-CAM uses the gradient information of the algorithm and could be used with any activation function and any architecture of CNNs.

## Discussion

This study reveals that a deep-learning algorithm, a powerful tool of artificial intelligence, can identify very delicate ECG changes when detecting MI. The performance for detecting MI was preserved with DLA using limb 6-lead ECG after adopting VAE, which reconstructed precordial 6-lead ECG from limb 6-lead ECG. To our knowledge, this is the first study that developed and validated a DLA for detecting MI using 12- and 6-lead ECGs.

As prompt detection and rapid management of MI is critical for reducing morbidity and mortality, more lives will be saved if MI can be monitored and detected in daily life through wearable or life type ECG devices. Because wireless transmission of ECG data through small portable devices is an existing technology, the current technical barrier remains the detection of myocardial infarction using the acquired ECGs. The conventional ECG interpretation method for diagnosing MI shows unsatisfactory performance, and its focus is to diagnose STEMI. Because the conventional interpretation method requires all the information from a 12-lead ECG, this method cannot be used in life type ECG equipment that uses limb 6-lead. In this study, we adopted artificial intelligence technology and developed a high-performance DLA for detecting all MI (including STEMI) using a 12-lead ECG. By adopting an VAE, which reconstructs precordial 6-lead ECG using limb 6-lead ECG, we achieved high performance for detecting MI using only a 6-lead ECG.

The most important aspect of deep learning is its ability to extract features and make an algorithm from various types of data, such as images, 2D data, and waveforms. Here, we used raw ECG data (2D numerical data, 12 × 4000) and interpreted ECG patterns for predicting cardiac arrest. Attia et al. developed deep-learning algorithms for screening cardiac contractile dysfunction, predicting the occurrence of atrial fibrillation during sinus rhythm, approximating age and sex, and detecting hyperkalemia using raw ECG data and demonstrated its feasibility^11,12,24,25^. Our study showed that a deep-learning-based algorithm using ECG could outperform cardiologists in diagnosing left ventricular hypertrophy and valvular heart disease^14,15,26,27^. We used a sensitivity map to visualize the regions of the ECGs that were used for decision-making by the DLA. The map shows that the DLA focused more on the ST segment to detect MI. The DLA also partially focused on the QRS complex and T-wave for detecting MI on a case-by-case basis. These findings have biological plausibility and are in agreement with current pathophysiologic knowledge, validating the importance of our findings.

One novel aspect of our study is the adoption of an VAE, that was used herein to develop an algorithm that was used to reconstruct the image and extract features from data. VAE is developed by unsupervised learning, and does not require labeling information^21^. That allowed us to take advantage of our large sample size, and develop an VAE with 412,461 ECGs that were not labeled for a specific disease. Although the performance of DLA with VAE using a 6-lead ECG is lower than that of DLA using a 12-lead ECG, it outperformed conventional interpretation using a 12-lead ECG. The finding that the performance of DLA with VAE was preserved in patients with LAD coronary lesion is interesting because, in current medical knowledge, MI with LAD coronary lesion could not be previously detected without the 6 precordial leads.

Medical data are almost imbalanced because there is a smaller proportion of patients with disease than the normal population. When developing an artificial intelligence model in the medical field, several methodologies have been used to amplify the data for the development dataset. In this study, we used several ECGs within 24 h of CAG in the development data. As the patients from the development, internal validation, and external validation dataset were exclusively divided, this does not affect the results of the validation dataset.

Our study has several limitations that warrant discussion and can be addressed in future studies. First, this was a retrospective study using data of conventional 12-lead ECGs. A prospective study specifically designed for confirming the accuracy of data from various wearable or portable ECG devices is warranted to apply the DLA to these devices. If we adopt DLAs in daily living, a study is also needed to confirm the performance at home and in other general environments and a strategy to manage false positives and negatives. As the clinical decision in the management of MI will always require human input, the study would require prospective validation with accurate clinical outcomes indicated by a blinded clinician with available clinical details and biochemical tests for clinical management. Second, we need to further explore the decision-making process of the DLA. For example, additional experiments are required to better understand the deep learning process and thereby understand which exact characteristics of the ST segment and QRS complex and the T-wave influence the algorithm’s decision. A considerable number of recent research have been dedicated to rendering artificial intelligence interpretable or explainable based on machine learning. In our next study, we adopted new technologies and enhanced interpretability in our prediction model using ECG^28^. This subject will be our next area of study, and it may become the new standard for discovering new medical knowledge about diseases and ECGs.

## Conclusion

The newly developed deep learning-based artificial intelligence algorithm demonstrated favorable performance in detecting MI using both 12-lead and 6-lead ECGs. These results suggest that MI could be detected with the use of wearable devices that integrates the artificial intelligence algorithm described in this work.

## Supplementary information

Supplementary Information.

## Abbreviations

2D: 2-Dimensional
CAG: Coronary angiography
CI: Confidence interval
CNN: Convolutional neural network
DLA: Deep learning-based artificial intelligence algorithm
ECG: Electrocardiography
Grad-CAM: Gradient-weighted class activation map
IHD: Ischemic heart disease
MI: Myocardial infarction
NPV: Negative predictive value
PPV: Positive predictive value
STEMI: ST-segment elevation myocardial infarction
VAE: Variational autoencoder
