# Supplementary material for: Artificial intelligence algorithm for detecting myocardial infarction using six-lead electrocardiography
Source: Sci Rep. 2020 Nov 24;10:20495. doi: 10.1038/s41598-020-77599-6 (PMC7686480; doi:10.1038/s41598-020-77599-6)
Supplement: Supplementary file 1 — Supplementary Information. [file 41598_2020_77599_MOESM1_ESM.pdf]

Supplemental material.
Title: Sensitivity maps of myocardial infarction electrocardiograms

Manuscript Title: Artificial intelligence algorithm for detecting myocardial infarction using six-lead electrocardiography
Manuscript Short Title: AI for detecting myocardial infarction

Younghoon Cho, MD MS[a,b]*, Joon-myoung Kwon, MD MS[c,d,e]*, Soohyun Cho, MD[a,b],
Kyung-Hee Kim, MD PhD[d,f], Jose R. Medina-Inojosa, MD, MSC[g], Soo Youn Lee, MD MS[d,f], Jinsik Park, MD PhD[e,f], and Byung-Hee Oh, MD PhD[f]

[a]Medical Research and Development Center, Bodyfriend, Seoul, South Korea;
[b]Medical Technology Labortory, Bodyfriend, Seoul, South Korea;
[c]Department of Emergency Medicine, Mediplex Sejong Hospital, Incheon, South Korea;
[d]Artificial Intelligence and Big Data Research Center, Sejong Medical Research Institute, Bucheon, South Korea;
[e]Medical research team, Medical AI, Seoul, South Korea;
[f]Division of Cardiology, Cardiovascular Center, Mediplex Sejong Hospital, Incheon, South Korea;
[g]Division of Preventive Cardiology, Department of Cardiovascular Medicine, Mayo Clinic, Rochester, MN, USA
*These two authors contributed equally to this work.

Corresponding Author:
Joon-myoung Kwon, MD, MS
Department of Emergency Medicine, Mediplex Sejong Hospital, Incheon, South Korea; dArtificial Intelligence and Big Data Research Center, Sejong Medical Research Institute, Bucheon, South Korea
20, Gyeyangmunhwa-ro, Gyeyang-gu, Incheon, Republic of Korea
Tel: 82-32-240-8129; Fax: 82-32-240-8094; E-mail: kwonjm@sejongh.co.kr

Kyung-Hee Kim, MD, PhD
Division of Cardiology, Department of Internal Medicine, Cardiovascular Center, Mediplex Sejong Hospital, Incheon, Korea
20, Gyeyangmunhwa-ro, Gyeyang-gu, Incheon, Republic of Korea
Tel: 82-32-240-8568; Fax: 82-32-240-8094; E-mail: learnbyliving9@gmail.com

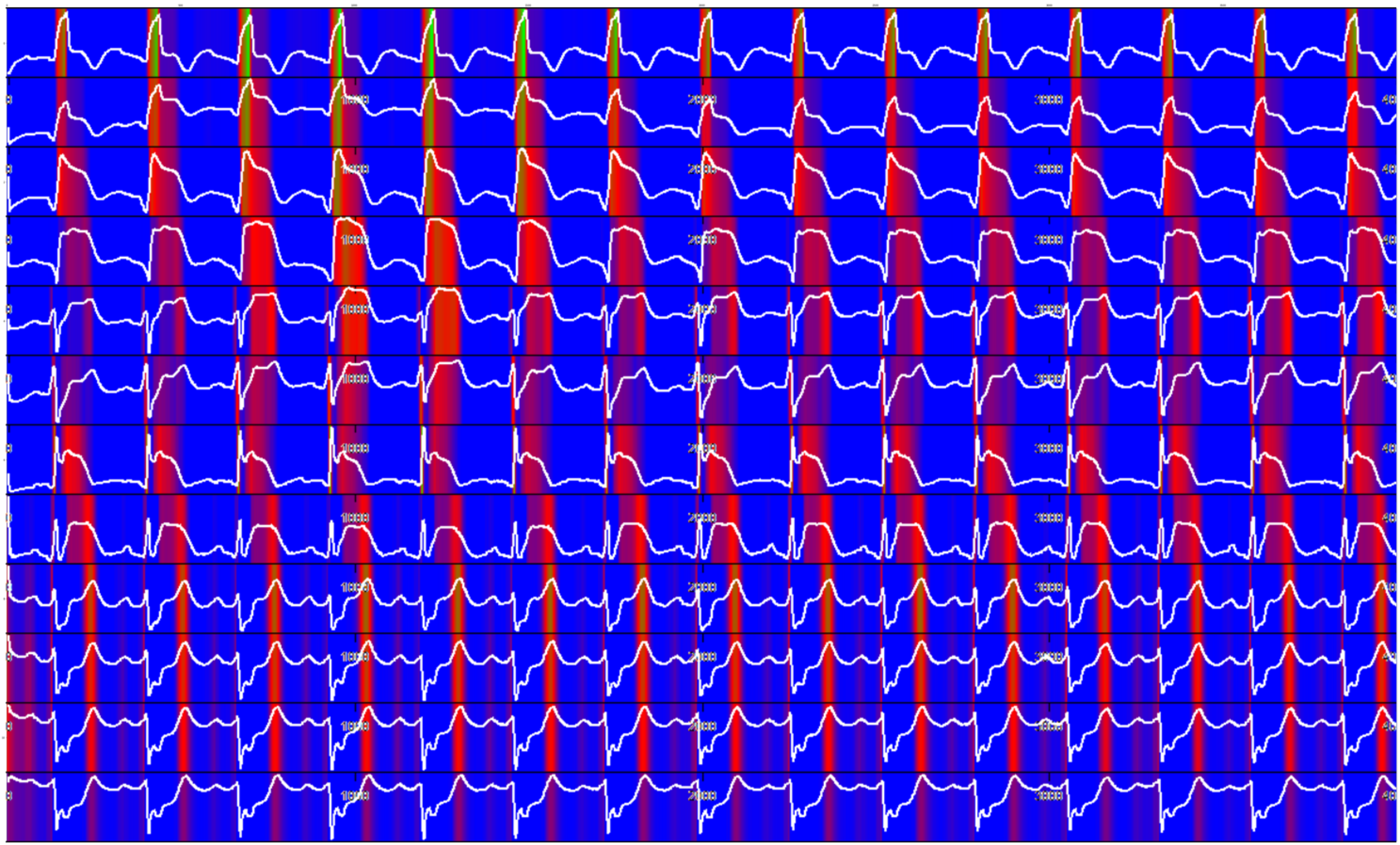

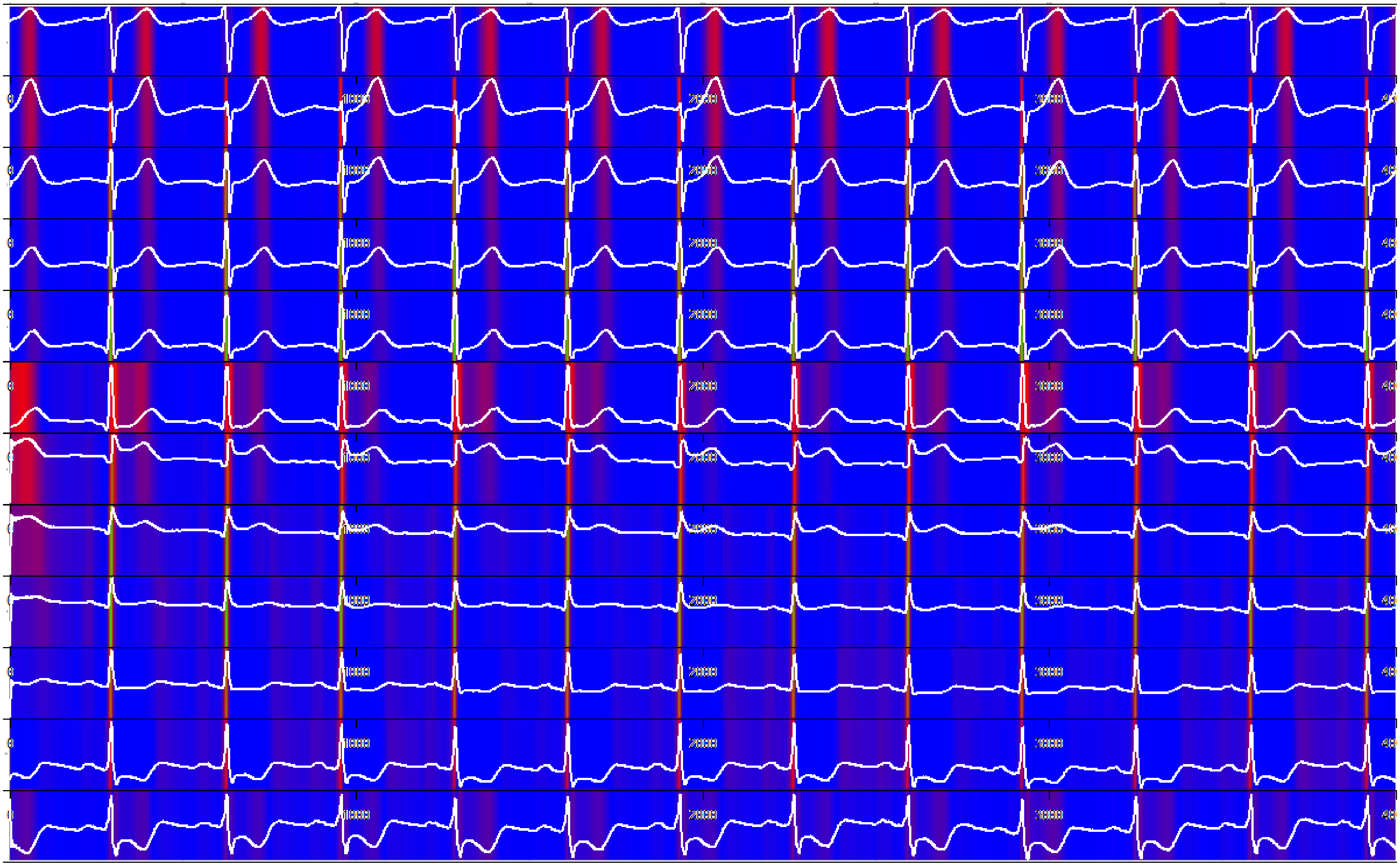

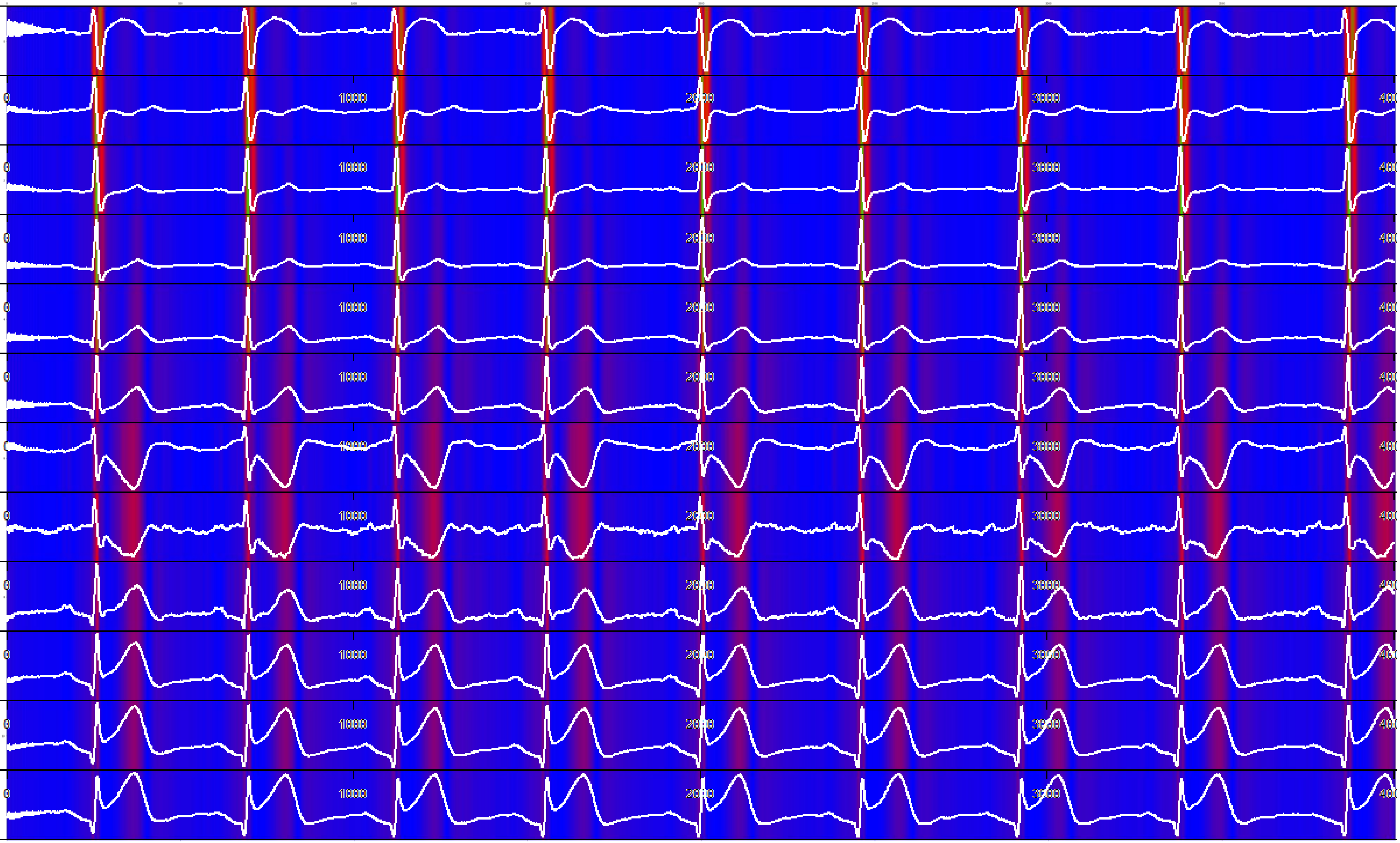

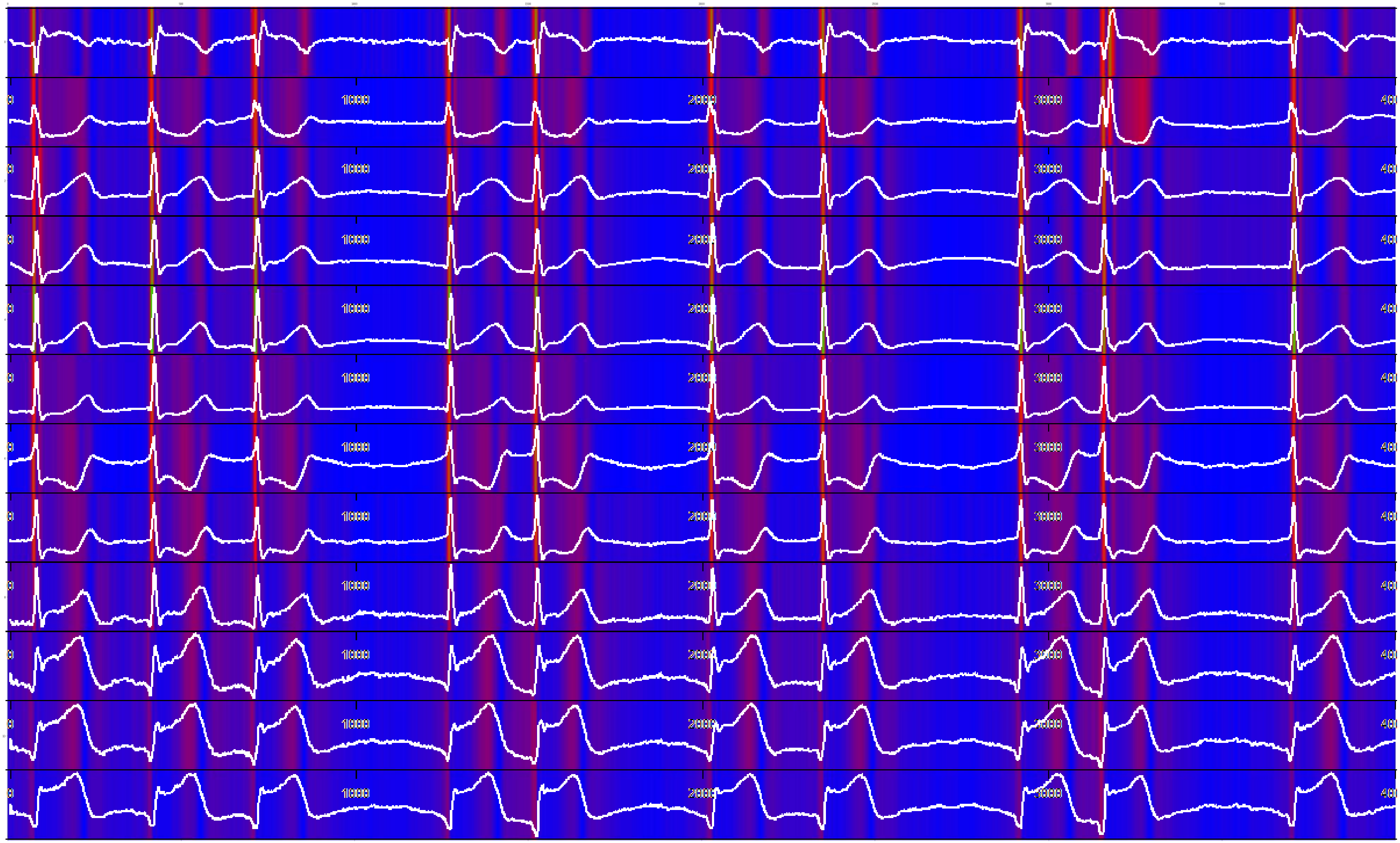

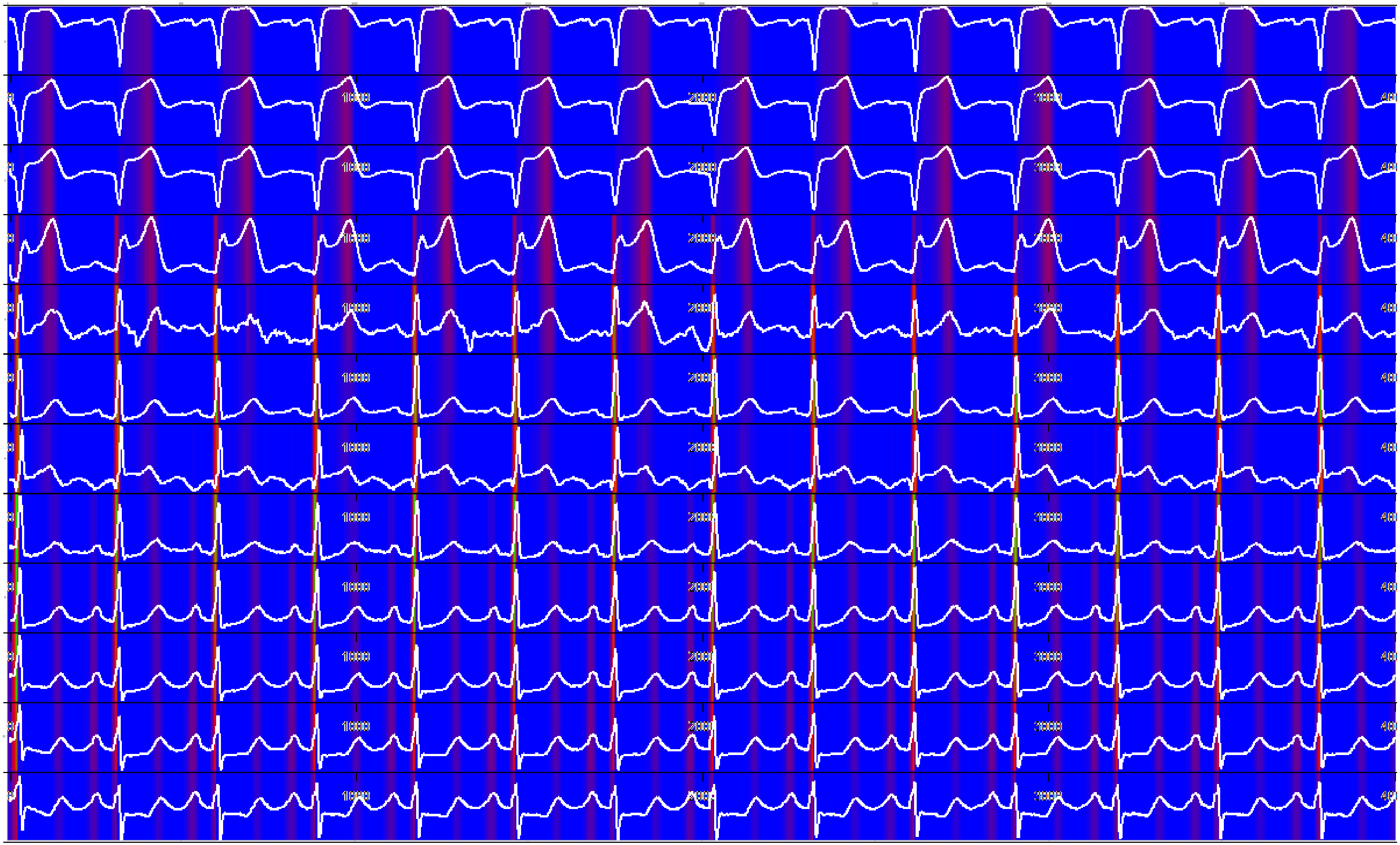

1000
2000
3000
